# Characterization of the Visceral Antinociceptive Effect of Glial Glutamate Transporter GLT-1 Upregulation by Ceftriaxone

**DOI:** 10.1155/2013/726891

**Published:** 2012-12-25

**Authors:** K. Roman, M. Yang, Robert L. Stephens

**Affiliations:** ^1^Department of Physiology and Cell Biology, The Ohio State University, 304 Hamilton Hall, 1645 Neil Avenue, Columbus, OH 43210, USA; ^2^Department of Gastroenterology, Daping Hospital, Third Military Medical University, Chongqing 400042, China

## Abstract

Recent studies demonstrate that glial glutamate transporter-1 (GLT-1) upregulation attenuates visceral nociception. The present work further characterized the effect of ceftriaxone- (CTX-) mediated GLT-1 upregulation on visceral hyperalgesia. Intrathecal pretreatment with dihydrokainate, a selective GLT-1 antagonist, produced a reversal of the antinociceptive response to bladder distension produced by CTX. The hyperalgesic response to urinary bladder distension caused by intravesicular acrolein was also attenuated by CTX treatment as was the enhanced time spent licking of abdominal area due to intravesicular acrolein. Bladder inflammation via cyclophosphamide injections enhanced the nociceptive to bladder distension; cohorts administered CTX and concomitant cyclophosphamide showed reduced hyperalgesic response. Cyclophosphamide-induced bladder hyperalgesia correlated with a significant 22% increase in GluR1 AMPA receptor subunit expression in the membrane fraction of the lumbosacral spinal cord, which was attenuated by CTX coadministration. Finally, neonatal colon insult-induced hyperalgesia caused by intracolonic mustard oil (2%) administration at P9 and P11 was attenuated by CTX. These studies suggest that GLT-1 upregulation (1) attenuates the hyperalgesia caused by bladder irritation/inflammation or by neonatal colonic insult, (2) acts at a spinal site, and (3) may produce antinociceptive effects by attenuating GluR1 membrane trafficking. These findings support further consideration of this FDA-approved drug to treat chronic pelvic pain syndromes.

## 1. Introduction

Interstitial cystitis/painful bladder syndrome (IC/PBS) is associated with several symptoms that include changes in bladder function and pain hypersensitivity that can develop spontaneously or be iatrogenically induced [1]. For example, the prolonged use of the chemotherapeutic drug cyclophosphamide leads to the induction of interstitial cystitis due to its breakdown in the liver to acrolein that is stored in the bladder and disrupts urothelial cell homeostasis [2]. Overall, therapeutic options for visceral pain disorders are limited largely to symptomatic relief [3]. Pain-transmitting afferent neurons utilize glutamate as the principle neurotransmitter to signal nociception related to the visceral organs [4]. Therefore, strategies to decrease spinal extracellular glutamate may be a viable option in pain management.

Glutamate transporters are essential for maintaining homeostatic levels of extracellular glutamate [5]. Previous studies show that inhibition of glutamate transporter activity in the spinal cord induces hyperalgesia and increases neuronal activity [6]. Reducing extracellular glutamate by over-expressing the predominant astrocytic glutamate transporter, GLT-1 is effective in animal models of both visceral [7–10] and neuropathic [11, 12] pain. Most of these studies used ceftriaxone (CTX), a substance that enhances GLT-1 expression and activity in rodents [9, 10].

This study will explore (1) the site of action of CTX to blunt the nociceptive response to bladder distension and (2) the effect of GLT-1 upregulation via CTX treatment on the enhanced nociceptive response in animal models of acute bladder irritation, chronic bladder inflammation, and neonatal colonic insult. Finally, these studies will explore the mechanism of the antinociceptive effect of CTX. The results reveal that pharmacological enhancement of GLT-1 expression via CTX treatment blunts the murine visceromotor response to bladder distension due to bladder irritation, bladder inflammation, and neonatal colonic insult. The antinociceptive effect is localized to the spinal cord and possibly the dorsal root ganglia, and reduced membrane trafficking of the AMPA subunit GluR1 may contribute to the antinociceptive effect. 

## 2. Methods

### 2.1. Animals

Two-month-old female FVB/N mice (20–25 g) were used for the experiments. Animals were housed on wood shavings with ad libitum access to food and water. The 12-hour light-dark cycle was maintained. Experiments were approved by the Institutional Animal Care and Use Committee from The Ohio State University and followed the guidelines of the Committee for Research and Ethical Issues of the International Association for the Study of Pain. 

### 2.2. Drug Administration

Doses (200, 100, or 50 mg/kg) of CTX was prepared in 0.9% saline and administered intraperitoneally (10 mL/kg; i.p.) at 10:00 am for seven consecutive days (1-week CTX); the control group received i.p. 0.9% saline injections. In one set of experiments, the selective GLT-1 antagonist dihydrokainate was administered (10 *μ*L) intrathecally (i.t.) at concentrations of 0.3, 0.03, or 0.003 mM one hour prior to urinary bladder distension, using a 28-gauge needle attached to a 10 *μ*L Hamilton syringe; the control group received i.t. 0.9% saline. Other sets of experiments examined the effect of 1-week CTX on (1) augmented visceromotor response to bladder distension produced by acute bladder irritation via intravesicular acrolein and (2) bladder inflammation produced by multiple injections of intraperitoneal cyclophosphamide over one week. In the acute bladder irritation model, animals were administered (under light isoflurane anesthesia) intravesicular acrolein (0.4 mM, 100 *μ*L) 1 hour before bladder distension was performed; the control group was administered intravesicular 0.9% saline. With the chronic bladder inflammation studies, animal cohorts received intraperitoneal injections of cyclophosphamide (80 mg/kg) or vehicle at day 0, 2, 4, and 6 [13]. All experiments were conducted on day 7. Finally, to study possible antinociceptive effects on neonatal stress-induced hyperalgesia, a 26-gauge microfil needle was attached to a 50 *μ*L Hamilton syringe and either 10 *μ*L of 2% mustard oil (Sigma, St. Louis, MO) or mineral oil (vehicle) was delivered intracolonically to mouse pups on postnatal days 9 and 11. To avoid sensitization of tissue surrounding the anus, Astroglide was applied liberally to the perianal region before needle insertion. Animals were observed briefly after intracolonic treatment (<5 min) for any damage to the tissue prior to being returned to the dam. All pups remained with dams until weaning on postnatal day 21. When the mice reached 7 weeks of age, 1-week prior to undergoing colorectal distension, animals received either 1-week CTX (200 mg/kg) or 1-week vehicle (0.9% saline) treatment. 

### 2.3. Electromyographic (EMG) Electrode Implantation

The golden standard for assessing visceral nociceptive response is by recording abdominal smooth muscle activity during colorectal distension [9]. Wires for electromyographic recording were surgically implanted in the external oblique muscle of mice [14]. In brief, after animals were anesthetized with i.p. ketamine (27.5 mg/kg; Hospira, Lake Forest, IL) and xylazine (10 mg/kg; Bayer, Shawnee Mission, KS) mice were shaven at the neck and the right side of the abdomen, the incision areas were disinfected, and a surgical incision (1 cm in length) was made in the back of the neck and lateral to the abdominal midline to expose the external oblique abdominal musculature. Then the electrode wires (Teflon coated stainless steel wire; Cooner Wire Sales, Chatsworth, CA) were sewn just above the inguinal ligament. The wires were then tunneled underneath the skin until they protruded on the back of the neck and sewn to the neck muscles. The neck and abdominal incisions are then closed using wound clips (Reflex 7 mm). To avoid fluid loss during surgery, animals were administered 0.1 mL of saline solution after completing the surgery. Animals were single housed and allowed to recover a minimum of 72 hours. Mice that develop motor defects, weight loss, or display stress-associated behaviors were excluded from the experiments.

### 2.4. Bladder Distension and Visceromotor Responses

 Bladder distension was performed as previously described [14]. On the day of the urinary bladder distension (bladder distension) experiments, mice were anesthetized with 4% isoflurane (induction) and 1.5% maintenance (Halocarbon Laboratories, River Edge, NJ), and a PE-10-tubing lubricated with Surgilube (E. Fougera, Melville, NY) was inserted (5–8 mm) transurethrally into the urinary bladder. The catheters were kept in place by taping the PE tubing to the base of the tail and secured to the urethral orifice with cyanoacrylate. Mice were placed in restraint devices (plastic semicircular tubing) while still sedated and allowed to recover and acclimate for a minimum of 1 hr before testing. Verification of proper placement of catheter was done after euthanasia. Visceromotor responses were quantified from the electromyographic (EMG) signals due to urinary bladder distension evoked musculature contractions as previously described [10]. Graded intensities of urinary bladder distension (0.05, 0.1, 0.15, and 0.2 mL normal saline) were used to compare the differences in the visceromotor response between 1-week vehicle and 1-week CTX treated animals. Electromyographic activity generated from the external oblique muscle (visceromotor responses), were recorded 10 seconds before bladder distension to establish baseline activity and 20 seconds during distension (response = increase above baseline), and 10 seconds after termination bladder distension. The EMG signal was then normalized as change over baseline using Spike 2 data acquisition software. 

### 2.5. Colorectal Distention (CRD)

CRD was performed as described previously [9]. Three days after EMG electrode implantation, the visceromotor response to colorectal distention was elicited by intracolonic balloon distention in triplicate at 15, 30, 45, and 60 mmHg pressure. The data were analyzed as the number of electromyographic spikes above baseline using Spike 2 software (CED, UK). 

### 2.6. Restraint Devices

Restraint devices were made from 50 mL conical tubes. An opening (~7 × 9 mm) was made at the superior aspect of the tube for access to the EMG recording electrodes. Small 0.5 cm diameter holes were made at the proximal end of the tubes to facilitate breathing. Also, a 25% portion of the conical tube was removed horizontally and a rectangular glass (2 × 5 cm) was glued in its place to accommodate the animal to a flat surface and avoid the rolling of the tube. The final restraining device holds a 17–27 g mouse. After the mouse was placed in the tube, the distal (open) end was secured with a gauze square and paper tape. The tube was then placed in a dark-colored cotton infant sock to reduce ambient light. The animals were allowed to acclimate inside the tube for 60 minutes before beginning recordings. The behavior of the mice before, during, and after distension was easily monitored by partial retraction of the sock. 

### 2.7. Abdominal Licking Response

An additional nociceptive response to bladder irritation was studied. Prior to beginning experiments animals were acclimatized to the cages in which observation of the animals occurred. After animals received 1-week CTX or vehicle (0.9% saline), on the day of the behavioral recordings, animals were lightly anesthetized with isoflurane and a 2 cm section of PE-10-tubing was lubricated with Astroglide jelly and placed inside the bladder via the urethra. A 1 cc syringe containing either 0.9% saline or acrolein (0.4 mM) was used to instill fluid (50 *μ*L) into the bladder over 1 minute through the catheter. Animals were placed in the observation chambers immediately after intravesicular treatment and allowed to acclimate for 30 minutes before initiating recording of time spent licking of abdominal area. All animals regained their righting reflex before commencing study (>5 minutes). Studies were conducted by blinded observers and treated groups were randomized. 

### 2.8. Spinal Cord Extraction and Subcellular Fractionation of Proteins

These procedures were performed as previously described by Park et al. with minor modifications [15]. Mice were euthanized by decapitation and the lumbosacral region of the spinal cord was excised and placed in a 5 mL tube containing 600 *μ*L of lysis buffer (10 mM Tris, 250 mM sucrose, 2 mM EGTA, 1 mM PMSF, 1 mM benzamidine, 2 mM leupeptin, 1 : 100 proteinase inhibitor cocktail, pH = 7.4) and homogenized via a tissue grinder (Wheaton, USA). Samples were centrifuged at 1,000 g for 15 minutes at 4°C. Afterwards, 20% of the supernatant containing the total protein (S1) was placed in a 1.5 mL tube and 80% of the remaining supernatant was centrifuged at 10,000 g for 20 min at 4°C. The supernatant containing the cytosolic fraction (S2) was collected and the remaining pellet was lysed in dH_2_O at 4°C and ultracentrifuged at 20,500 g for 30 minutes at 4°C. The supernatant was discarded and the remaining pellet containing the synaptosomal membrane was collected and dissolved in modified 1X lysis (from stock) buffer (10 mM Tris, 250 mM sucrose, 2 mM EGTA, 1 mM PMSF, 1 mM benzamidine, 2 mM leupeptin, 1 : 100 proteinase inhibitor cocktail, 2% SDS, 0.1% Triton ×100, in 7.99 mL of dH_2_O, pH = 7.4). The pellet is sonicated with five quick bursts. Protein concentration of S1, S2, and P3 was determined with a Coomassie (Bradford) protein kit assay (Thermo Scientific). 

### 2.9. Western Blot

 Protein samples were extracted from the lumbosacral spinal cord. The desired final protein concentration (60 *μ*g) was loaded in 8% SDS-PAGE gel, and then electroblotted onto nitrocellulose membrane using a mini gel and mini transblot apparatus (Bio-Rad laboratories, cat.170-3935). The membranes were then rinsed and blocked with 3% nonfat milk in TBST buffer (0.1% Tween 20, 20 mM Tris, 137 mM NaCl; pH = 7.6) at room temperature for 1 hour. The membranes were then incubated with the primary rabbit polyclonal antiglutamate receptor 1 (1 : 1,000; Millipore; AB1504) in 3% milk-TBST buffer overnight at 4°C. Afterwards, the membranes were exposed to the secondary antibody goat anti-rabbit IgG with horseradish peroxidase (HRP) in 3% milk-TBST buffer (1 : 3,000 dilution) for 1.5 hours at room temperature. Amersham ECL western blotting reagents (GE Healthcare; lot. 4624547) were used to detect HRP-antibody signal. The nitrocellulose membranes were rinsed with stripping buffer (0.2 M glycine and 0.05% Tween 20; pH = 2.5) for 45 minutes, washed (TBST), and placed in blocking buffer (3% nonfat milk) at room temperature for 1 hour. The membranes were relabeled with rabbit polyclonal anti-*β*-actin (1 : 1,000; Santa Cruz; sc-130656). Then, the membranes were exposed to the secondary antibody goat anti-rabbit IgG with horseradish peroxidase (1 : 3,000 dilution; Bio-Rad; cat.170-6515) in 3% milk-TBST buffer for 1.5 hours. Amersham ECL western blotting reagents (GE Healthcare; lot. 4624547) were used to detect HRP-antibody signal. 

### 2.10. Statistical Analysis

All elicited EMG signals were rectified and the area under the curve (AUC) for the 20-second distension was subtracted from the AUC of the 10-second baseline, as previously described [14]. Regarding the western blotting data, Bio-Rad Quantity One software was used to determine optical density of western blots. Protein blots were analyzed by standardizing the optical density of GluR1 against the optical density of the *β*-actin protein. The quantitative data in each study was expressed as mean ± SEM. For behavioral experiments, a one way or two way ANOVA, followed by least significant difference (LSD) multiple comparison post hoc tests, was performed as appropriate; data were considered statistically significant, different if *P* < 0.05.

## 3. Results

### 3.1. Intrathecal Dihydrokainate (DHK) Reversed CTX-Attenuated Visceromotor Response to Urinary Bladder Distension

**Figure 1:**
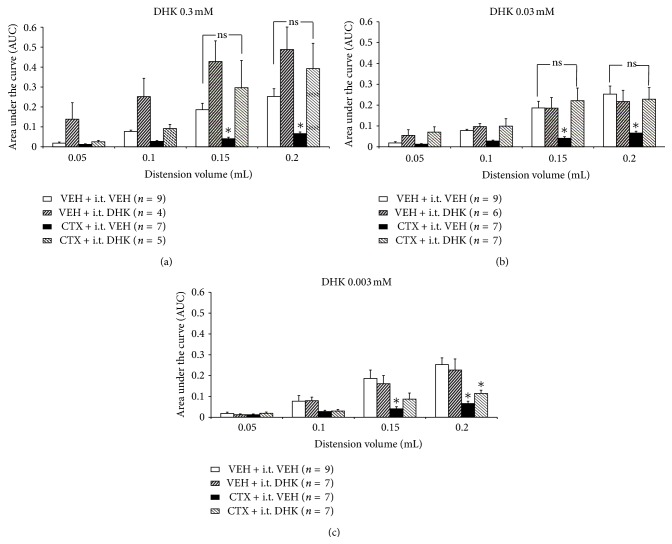
(a)–(c) Intrathecal injection of dihydrokainate (DHK), a selective GLT-1 antagonist, reversed the attenuated visceromotor response induced by 1 wk ceftriaxone (CTX) at 0.3 mM and 0.03 mM, but not the 0.003 mM dose. (a) Mice that received 1 wk VEH + i.t. DHK (0.3 mM) showed an elevated visceromotor response relative to control (1 wk VEH + i.t. VEH). Treatment with i.t. DHK (0.3 mM) reversed the CTX-attenuated visceromotor response. (b) Mice treated with 1 wk VEH + i.t. DHK (0.03 mM) showed no difference in the visceromotor response relative to control (*P* > 0.05). Treatment with i.t. DHK (0.03 mM) reversed the CTX-attenuated visceromotor response. (c) In contrast, mice administered 1 wk CTX + i.t. DHK (0.003 mM) showed no reversal of the attenuated visceromotor response produced by 1 wk CTX + i.t. VEH.

Given that bladder distension-evoked visceromotor responses are reduced in CTX treated mice and mediated by GLT-1 upregulation [10], this experiment is designed to investigate the anatomical site of action. A selective GLT-1 antagonist, dihydrokainate, was administered intrathecally (10 *μ*L) at different concentrations (0.3, 0.03, and 0.003 mM) to assess if GLT-1 upregulation at the spinal cord or dorsal root ganglia is responsible for mediating the reduced visceromotor response to urinary bladder distension. The results show that animals treated with 1 wk CTX (200 mg/kg) + i.t. VEH had a significant 62–70% decrease in the visceromotor response to 0.15 and 0.2 mL bladder distension volumes compared to animals treated with 1 wk VEH + i.t. VEH (Figures 1(a)–1(c)). The highest intrathecal DHK dose tested (1 wk VEH + i.t. DHK at 0.3 mM) reversed the CTX-mitigated response, but also produced an increased visceromotor response (93–123% at 0.15 and 0.2 mL) compared to the control group (1 wk VEH + i.t. VEH) (Figure 1(a)). To determine if i.t. DHK reverses the effect of GLT-1 upregulation at a dose not affecting control responses, a tenfold lower dose of DHK (0.03 mM) was administered intrathecally and the visceromotor response to bladder distension was recorded. 1 wk VEH + i.t. DHK 0.03 mM treatment did not significantly alter the visceromotor response to graded bladder distension (*P* > 0.05) compared to control (1 wk VEH + i.t. VEH) cohorts (Figure 1(b)). However, the cohort treated with 1 wk CTX + i.t. DHK at 0.03 mM showed a reversal of the attenuated visceromotor response produced in the 1 wk CTX + i.t. VEH cohort at 0.15 and 0.2 mL (Figure 1(b)). Intrathecal administration of an additional tenfold lower dose of i.t. DHK (0.003 mM) was ineffective to reverse the effect of 1 wk CTX on the visceromotor response to bladder distension of 0.15 and 0.2 mL (Figure 1(c)), suggesting a dose response relationship. Thus, intrathecal injection of selective GLT-1 antagonist DHK reversed the antinociceptive effects of 1-week CTX administration. 

### 3.2. CTX Dose Ineffective to Alter the Normal Visceromotor Response to Urinary Bladder Distension Attenuates Irritant-Induced Visceral Hyperalgesia

**Figure 2:**
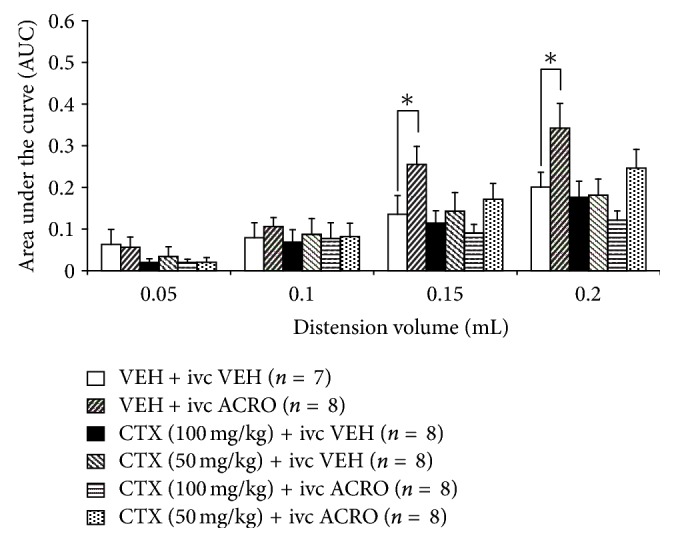
Lower doses of CTX (50 mg/kg or 100 mg/kg × 7 days) did not produce a visceromotor response different from the control group at graded distension volumes. Animals administered 1 wk VEH + intravesicular acrolein (ivc ACRO) showed a significant increase in the visceromotor response compared to the control group at 0.15 and 0.2 mL (∗*P* < 0.05). In contrast, mice treated with 1 wk CTX (50 or 100 mg/kg) + ivc ACRO did not show an enhanced visceromotor response to bladder distension compared to the 1 wk VEH + ivc VEH treated cohort at 0.15 and 0.2 mL.

Since previous work showed that 1 wk CTX at a 200 mg/kg dose attenuates the normal visceromotor response to urinary bladder distension [10], a salient issue is the efficacy of CTX doses not causing reduced visceromotor response. Thus, animals received daily systemic injection of either 100 mg/kg or 50 mg/kg of CTX for 1-week. One hour before graded urinary bladder distension bladder irritation was induced, bladder irritation was induced by intravesicular (ivc) acrolein (ACRO; 0.4 mM). Cohorts treated with 1 wk VEH + ivc ACRO showed a significant increase (70–90% at 0.15 and 0.2 mL; ∗*P* < 0.05) in visceromotor response compared to 1 wk VEH + ivc VEH (Figure 2). CTX at doses not affecting the normal visceromotor response (50 and 100 mg/kg for 1-week) both attenuated the visceral hyperalgesia caused by intravesicular acrolein at 0.15 and 0.2 mL (Figure 2).

### 3.3. Abdominal Licking Behavior after Acute Bladder Irritation Is Reduced after 1-Week CTX

**Figure 3:**
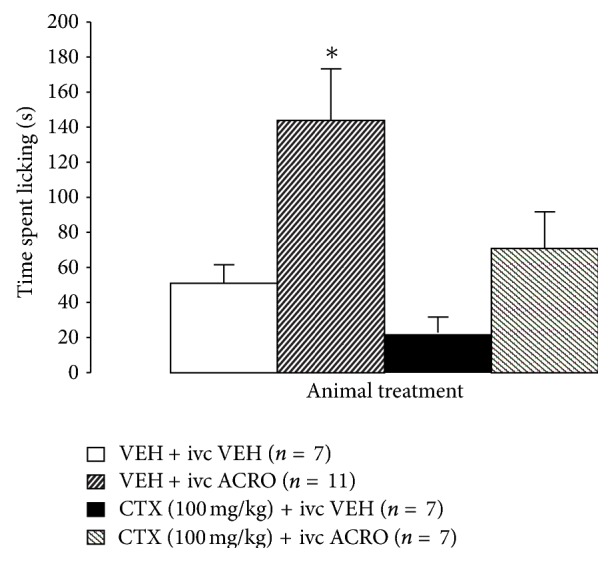
Mice treated with vehicle or CTX (100 mg/kg) for seven days were observed for the amount of time spent licking the abdominal area for 1 hr after receiving intravesicular (ivc) vehicle or acrolein (0.4 mM). The data show a significantly enhanced time spent licking in animals that received 1 wk VEH + ivc ACRO, compared to the control group (1 wk VEH + ivc VEH; ∗*P* < 0.05). This enhanced nociceptive response was attenuated in cohorts receiving 1 wk CTX + ivc ACRO treatment.

To confirm that CTX administration reverses visceral nociceptive behavior in rodents, an additional visceral pain model was studied. In 1 wk CTX (100 mg/kg) or vehicle treated cohorts, the bladder was infused (50 *μ*L) with either acrolein (0.4 mM) or saline 0.9% over a 1-minute period, under light isoflurane anesthesia. After a 30-minute recovery time, the number of abdominal licks were quantified for 1 hour. Abdominal licking was increased 64% in the 1 wk VEH + ivc ACRO, cohort compared to control (1 wk VEH + ivc VEH). In contrast, animals treated with 1 wk CTX + ivc ACRO did not show an enhanced time spent licking of the abdomen compared to 1 wk VEH + ivc VEH (Figure 3). 

### 3.4. Effect of CTX on Chronic Bladder Inflammation-Induced Changes in the Visceromotor Response to Bladder Distension

**Figure 4:**
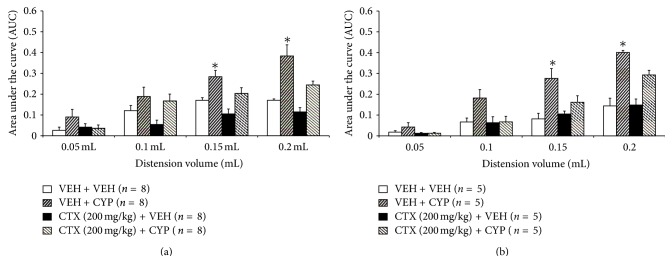
(a)-(b) Cyclophosphamide (1 wk VEH + 1 wk CYP) significantly increased the visceromotor to urinary bladder distension at 0.15 and 0.2 mL, compared to 1 wk VEH + 1 wk VEH controls. (∗*P* < 0.05). (a) The enhanced visceromotor response produced in the 1 wk VEH + CYP group was attenuated by 1 wk daily CTX administration (1 wk CTX (200 mg/kg) + 1 wk CYP). (b) Administration of a lower dose of CTX (100 mg/kg; i.p.) for seven days also attenuated the enhanced visceromotor response to bladder distension of mice treated with cyclophosphamide (80 mg/kg) during the 7-day period prior to undergoing bladder distension (∗*P* < 0.05).

Previous studies on rodents showing that repeated injections of cyclophosphamide over 1-week leading to prolonged bladder inflammation reproduce some aspects of interstitial cystitis [2, 16, 17]. To further explore if CTX produces a reduction of visceromotor response to bladder distension in this model, mice received one-week CTX (200 mg/kg) treatment at the onset of induced inflammation of the bladder (at day 0) elicited by i.p. injection of cyclophosphamide (80 mg/kg; days 0, 2, 4, and 6). The results indicate that the 1 wk VEH + cyclophosphamide cohort showed an enhanced visceromotor response to bladder distension compared to 1 wk VEH + VEH treated group (66%–120% at 0.15 and 0.2 mL) (Figure 4(a)). 1 wk CTX administered to cyclophosphamide-treated animals blunted the enhanced visceromotor response to bladder distension seen in the 1 wk VEH + cyclophosphamide cohort (Figure 4(a)). Thus, the data suggests that GLT-1 upregulation by 1 wk CTX attenuates bladder inflammation-induced hyperalgesia.

Animals administered a lower dose of CTX (100 mg/kg; ip) for seven days beginning at day 0 and did not elicit a visceromotor response different from 1 wk VEH + 1 wk VEH treated cohorts after graded urinary bladder distension (Figure 4(b)). As seen before, the 1 wk VEH + 1 wk cyclophosphamide group produced a significantly enhanced visceromotor response to bladder distension (178–239% at 0.15 and 0.2 mL) compared to controls. However, mice treated with 1 wk CTX (100 mg/kg) + cyclophosphamide showed no enhanced visceromotor response compared to 1 wk VEH + 1 wk VEH controls (Figure 4(b)). 

### 3.5. GluR1 Trafficking and Chronic Bladder Nociception

**Figure 5:**
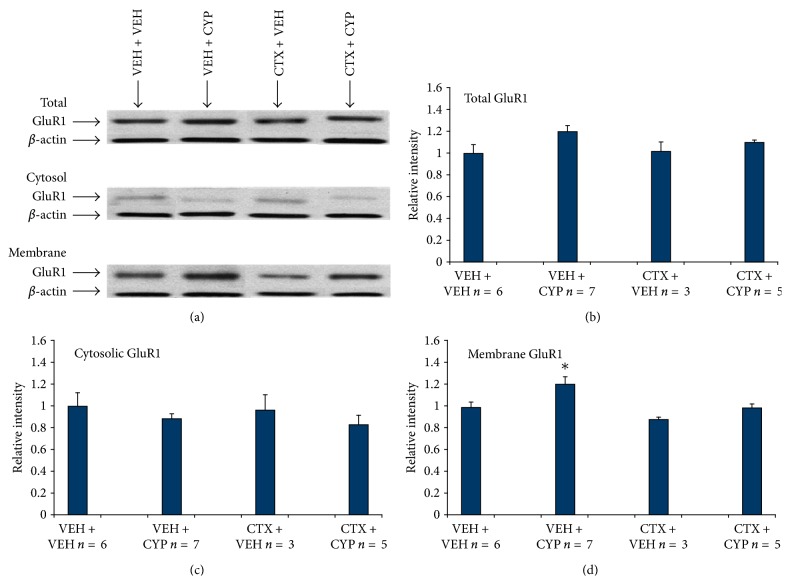
(a)-(b) Total and membrane-associated GluR1 is increased after 1-week cyclophosphamide treatment. Enhanced GluR1 membrane trafficking is mitigated after concomitant 1 wk CTX and cyclophosphamide treatment. (a) Western blot comparing Total, cytosolic, and membrane GruR1 expression. (b) Representative western blot densitometry shows a 20% increase in total GluR1 expression in 1 wk VEH + CYP and a 22% increase in the membrane compared to control cohorts 1 wk VEH + VEH (∗*P* < 0.05).

Studies were initiated to explore the putative molecular mechanism of antinociceptive action of CTX downstream from reduction of extracellular glutamate. Altered AMPA subunit trafficking has been proposed to mediate hyperalgesia [18, 19]. The lumbosacral region of the spinal cord showed a 20% increase in total GluR1 expression after 1 wk VEH + cyclophosphamide treatment compare to control 1 wk VEH + VEH cohorts (Figures 5(a)-5(b)). This total increase in GluR1 expression after 1 wk VEH + cyclophosphamide parallels the increased trafficking to the membrane, which showed a significant 22% increase in GluR1 expression (Figure 5(d)). However, concomitant CTX administered with cyclophosphamide over 1 week attenuated this enhanced GluR1 membrane trafficking (Figure 5(d)). There was no significant difference in cytosolic GluR1 trafficking between any of the treated groups and the control 1 wk VEH + VEH cohort (Figure 5(c)). 

### 3.6. GLT-1 Upregulation Mitigates the Enhanced Visceromotor Response in Adult Mice after Neonatal Intracolonic Mustard Oil Administration

**Figure 6:**
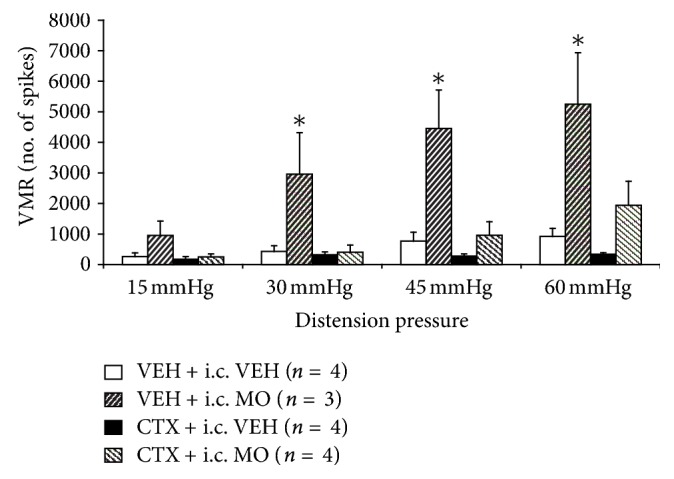
Neonatal stress-induced visceral hypersensitivity in adult mice. Postnatal day 9 and 11 treatment with intracolonic 2% mustard oil (1 wk VEH + i.c. MO) dramatically increased the visceromotor response to colorectal distension at 30–60 mm Hg in adult mice, compared to control (1 wk VEH + i.c. VEH) cohorts. In marked contrast, ceftriaxone administered 1-week before colon distension (1 wk CTX + (i.c.) MO) completely attenuated the enhanced visceromotor response to colorectal distension produced by neonatal colon irritation (∗*P* < 0.05).

 Early adverse events have been identified as an important etiological contributor to chronic visceral pain [20, 21]. Recent preclinical studies suggest that early life stress or visceral organ insult induces long-term visceral hypersensitivity [22, 23]. This experiment was designed to assess whether *therapeutic* GLT-1 upregulation via 1-week CTX can attenuate visceral hyperalgesia produced by neonatal intracolonic mustard oil administration. The results show that animals that received 2% mustard oil (MO) on postnatal days 9 and 11 produced a markedly enhanced visceromotor response to CRD at 8 weeks of age at all distension pressures compared to 1 wk VEH + intracolonic VEH cohorts (Figure 6). Enhanced responses were three- to fivefold higher than the response produced by intracolonic vehicle administration (1 wk VEH + i.c. VEH). One-week treatment with CTX remarkably attenuated the hyperalgesia caused by neonatal intracolonic mustard oil. Thus, therapeutic treatment with CTX was quite effective at mitigating visceral hypersensitivity caused by noxious neonatal colonic insult.

## 4. Discussion

The principle findings of these studies were (1) reduced visceromotor response to urinary bladder distension after GLT-1 up-regulator CTX administration was reversed by intrathecal dihydrokainate (selective GLT-1 antagonist), (2) CTX mitigated the visceromotor response to urinary bladder distension after acute bladder irritation or inflammation, and (3) *therapeutic* CTX administration attenuates neonatal colonic insult-induced adult visceral hyperalgesia.

These findings support a potential translational approach of GLT-1 upregulation to reduce visceral pain. Pharmacologically enhanced GLT-1 expression provides a viable strategy to reduce extracellular glutamate and mitigate visceral nociception via spinal or peripheral mechanisms. Intrathecal DHK (*k*_*i*_ = 23 *μ*M) administered intrathecally at a (0.03 mM (30 *μ*M)) concentration reversed the CTX effect without affecting the normal visceromotor response to urinary bladder distension. Hence, the effectiveness of intrathecal DHK suggests that the spinal cord and/or dorsal root ganglia (DRG) may be site(s) of action of GLT-1 upregulation to mitigate the visceral nociceptive response in both bladder and colon [8]. The same study suggests that the hindbrain is not an antinociceptive site of action of CTX in colon distension models [8]. 

Recent studies suggest that strategies to reduce extracellular glutamate in the trigeminal nuclei and the DRG have antinociceptive effects [24, 25]. Xie et al. (2012) showed that local riluzole, a glutamate reuptake activator/sodium channel blocker, administration for seven days to the L5 spinal nerve after DRG inflammation suppressed mechanical pain behavior via reduced spontaneous A*αβ* cell activity in rats [25]. Thus, peripheral sensory ganglia may also be a site of action that mediates CTX antinociceptive effects [24]. Glutamate transporters GLT-1, GLAST, and EAAC1 are found in DRG satellite cells [26, 27]. The ability of CTX or dihydrokainate to alter extracellular glutamate levels via satellite cells is unknown. Thus, further studies are implicated to determine the role of glutamate transporters regulating extracellular glutamate in DRG mediating visceral or somatic nociception. This intriguing possibility is worthy of further exploration. 

Evidence suggests that the antinociceptive mechanism of action of CTX is the enhancement of glutamate transporter GLT-1 activity and not probiotic or other effects [8]. The EC_50_ required to increase GLT-1 expression by CTX is 3.5 *μ*M [28]; blood levels after 200 mg/kg dose of CTX reach likely millimolar concentrations. In vivo, CTX treatment for five days at 100 but not 50 mg/kg significantly reduced the extracellular glutamate concentrations in the nucleus accumbens [29] suggesting enhanced glutamate uptake activity. New findings in the present report show that lower doses of 1 wk CTX (100 mg/kg or 50 mg/kg) not affecting the normal visceromotor response to urinary bladder distension effectively mitigated the enhanced visceromotor response to organ distension after bladder irritation (Figure 2). Moreover, 1 wk CTX (100 mg/kg) reduced abdominal licking response after acute bladder irritation (Figure 3). Thus, doses of CTX not affecting normal visceromotor response to bladder distension were effective to mitigate bladder hypersensitivity due to locally activated bladder sensory mechanisms.

 Previous murine studies showed that four intraperitoneal injections of cyclophosphamide over 1 week induce prolonged (chronic) bladder inflammation that models aspects of interstitial cystitis [13, 30]. Cyclophosphamide is metabolized into acrolein, an irritant, and it is concentrated in the bladder. The present study revealed that prolonged bladder hypersensitivity increases GluR1 membrane trafficking by 22% (Figures 5(a)-5(b)). The significant enhancement of GluR1 expression in the membrane suggests sustained GluR1 trafficking after bladder inflammation may contribute to the maintenance of central sensitization. One-week CTX treatment diminished these enhancements (total GluR1 and trafficking of GluR1 to the membrane) (Figures 5(a)-5(b)), in concert with its antinociceptive effects (Figures 5(a)-5(b)). This supports the notion that upregulation of GLT-1 may relieve pain by limiting GluR1 membrane trafficking that contributes to enhanced sensory signaling of second-order neurons. Ionotropic and metabotropic glutamate receptors play an important role in modulating spinal neuronal activity. In particular, studies have shown the importance of AMPA receptors in establishing central sensitization [31]. Since AMPA-mediated cation influx in the spinal cord is regulated by glutamate binding and increased expression of GluR1 subunits contributes to increased calcium permeability [32] due to the internalization of GluR2 subunits [15] changes in GluR1 trafficking become important in studies of pain [19]. Studies have demonstrated that enhanced GluR1 trafficking takes place in somatic inflammatory pain [33], but this has not been well studied in visceral pain. Galan et al. (2003) showed that GluR1 trafficking is significantly enhanced after acute capsaicin administration to the colon [34]. Therefore, the antinociceptive effect of CTX in prolonged visceral inflammation models may be due to hindrance of glutamate receptor activation and postsynaptic events (AMPA subunit trafficking, activation of MAP kinase pathway [35]). 

Epidemiological data have shown that a history of early adverse life events in the form of emotional, sexual, or physical abuse is a major predisposing factor for the development of functional visceral pain syndromes later in life [21, 36]. Models inducing neonatal perturbations resulting in enhanced adult visceral hyperalgesia have been developed in rodents [37] and include induction of neonatal intracolonic irritation [22, 38]. Thus, perturbation of intestinal homeostasis in early life appears to result in permanent central sensitization possibly driven by an early increase in growth factor expression and maintained by permanent changes in TRPA1 function in DRG neurons [22]. Given the marked effectiveness of CTX to mitigate hyperalgesia after neonatal colon irritation (Figure 6), it would be important to examine the effect of GLT-1 upregulation on other models of early-life or adult noxious events that lead to visceral hyperalgesia or allodynia.

In sum, these studies suggest that GLT-1 upregulation produced by CTX, acting at the spinal cord or DRG, reduced the visceromotor response to urinary bladder distension and hyperalgesia caused by bladder irritation/inflammation and hyperalgesia in adult mice after neonatal colon insult. Given that ceftriaxone is an FDA-approved drug and it is in phase III clinical trial for amyotropic lateral sclerosis (Clinicaltrial.gov, identifier NCT00349622), this treatment may comprise an important addition to the available treatments for visceral pain disorders.
